# Analysis of pupillometer results according to disease stage in patients with Parkinson’s disease

**DOI:** 10.1038/s41598-021-97599-4

**Published:** 2021-09-09

**Authors:** Sooyeoun You, Jeong-Ho Hong, Joonsang Yoo

**Affiliations:** 1 Department of Neurology, Dongsan Hospital, Keimyung University School of Medicine, Daegu, Republic of Korea; 2 Department of Neurology, Yongin Severance Hospital, Yonsei University College of Medicine, 363 Dongbaekjukjeon-daero, Giheung-gu, Yongin-si, Gyeonggi-do 16995 Korea

**Keywords:** Diseases, Neurology, Signs and symptoms

## Abstract

We performed pupillometer testing on 132 patients with Parkinson’s disease, stratified into two groups according to the disease stage. Neurological examinations and pupillometry were performed in the ON state. Patients in the Hoehn and Yahr stages 1 and 2 comprised the early group, and patients in stages 3–5 formed the late group. We performed age- and sex-matched (2:1) propensity score matching to compensate for the effect of age on pupil light reflex. Eight pupillometer parameters were measured and compared between the two groups. After the propensity score matching, the early group had 64 patients and the late group had 32 patients. The late group had a longer disease duration and took a higher levodopa equivalent dose than the early group. The constriction velocity (*P* = 0.006) and maximum constriction velocity (*P* = 0.005) were significantly faster in the early group than in the late group. Pupil size, minimum diameter, and dilation velocity were similar in both groups. The pupillary contraction velocity decreased with the disease progression, suggesting that the progression of Parkinson’s disease could be identified by the pupil constriction velocity.

## Introduction

The pupil light reflex (PLR) refers to the process of constriction and subsequent dilation of the pupil. It plays a major role in determining the retinal image quality and response to light. PLR is also an important measure of the autonomic nervous system function. Sphincter and dilators that are involved in modulating the PLR are innervated by parasympathetic and sympathetic nerves; therefore, several PLR parameters can be used as indicators of either sympathetic or parasympathetic modulation. The measurement of PLR is a non-invasive method to determine the parasympathetic and sympathetic balance, and is a useful test for patients with neurological disorders. Factors affecting the average pupil diameter include age, sex, colors of the iris, optical media clarity, retinal and optic nerve health, and ambient light level^1^.

Automated pupillometry is currently used to monitoring the nervous system functions of critically ill patients^2^. In particular, the ability to measure pupillary function in a rapid, non-invasive, reliable, and quantifiable manner is of great help in the clinical diagnosis of critically ill patients in neuro-intensive care units. In addition, patients with neurodegenerative diseases, such as Alzheimer's disease (AD) and Parkinson's disease (PD), exhibit PLR abnormalities due to cholinergic hypofunction^3,4^. In previous studies, compared with healthy age-matched subjects, patients with AD or PD, had significantly lowered maximum constriction velocity and maximum constriction amplitude, among the other components of PLR^5,6^.

In previous studies on patients with PD, pupillometry results were helpful in detecting subclinical autonomic dysfunction; however they did not show a correlation with disease duration, the motor section of the Unified Parkinson’s disease rating scale (UPDRS), or with the presynaptic dopaminergic dysfunction^6,7^. In this study, we investigated whether there is a relationship between the disease stage and pupillometry findings in PD patients.

## Methods

This study was conducted by retrospectively reviewing the data of consecutive patients with PD who visited the outpatient department of neurology at Dongsan Medical Center between September 2019 and January 2020. This retrospective study was approved by the Institutional Review Board of Dongsan Hospital (IRB No. 2020-05-012) and requirement for informed consent was waived due to its retrospective nature. Our study was implemented in accordance with the ethical standards of the 1964 Declaration of Helsinki and its later amendments.

### Participants

PD was diagnosed according to the Movement Disorder Society (MDS) PD Criteria^8^, and the examination was conducted by a movement specialist (S.Y.). We excluded patients with Parkinson-plus syndromes, such as multiple system atrophy, progressive supranuclear palsy, and corticobasal degeneration; patients diagnosed with vascular parkinsonism, drug-induced parkinsonism, AD, and other neurodegenerative diseases; patients with known ocular or systemic diseases, such as glaucoma, corneal damage, and uncontrolled diabetes mellitus, which could affect the PLR; and users of ocular topical agents with sympathomimetic or parasympathomimetic effects. However, patients taking drugs with systemic anticholinergic effects (procyclidine and trihexyphenidyl) were not excluded.

Neurological examinations were performed in the ON state and de novo patients were excluded from this study. We also investigated the patients’ disease durations and levodopa equivalent daily doses (LEDD)^9^. The disease stage of the patients was measured using the Hoehn and Yahr (HY) rating scale. Patients belonging to HY stages 1 and 2 were classified as early stage, and those belonging to stages 3–5 were classified as late stage.

### Pupillometry

The PLR was measured in both the eyes using a fully automated pupillometry system (NPi^®^-200 Pupillometer, Neuroptics, CA), and eight parameters (neurological pupilar index [NPi], pupil size, maximum diameter, changing ratio of pupil size, constriction velocity, maximum constriction velocity, latency of constriction velocity, and dilation velocity) were obtained for each eye. For each variable, the mean value measured in both the eyes was used. PLR measurements were performed concurrently with the patients’ neurological examinations, and tests were always performed at the same locations at a constant temperature and brightness.

### Statistical analysis

Data are expressed as mean ± standard deviations, medians (interquartile range), or numbers (percentages), as statistically appropriate. We compared the pupillometric parameters between patients from early and late HY stages using Chi-square tests, Wilcoxon rank-sum tests, or Student’s *t*-test. To reduce the effects of aging on the pupillometric parameters^10,11^, we performed propensity score matching. The propensity score was calculated using age and sex. After estimating the propensity score, the early and the late HY stage groups were matched at a ratio of 2:1. All *P*-values were two-tailed, and variables were considered significant at *P* < 0.05. All statistical analyses including the matching process were performed using R software version 4.0.3 (R Foundation for Statistical Computing, Vienna, Austria. http://www.R-project.org).

## Results

### Before propensity score matching

**Figure 1:**
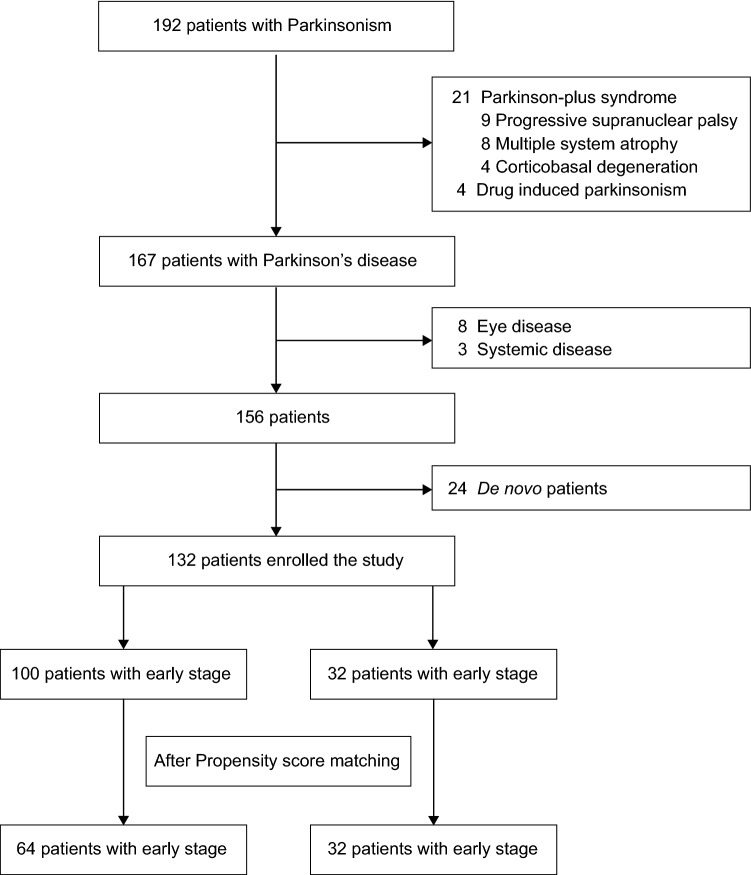
Participants flow chart. The flowchart demonstrates the process of analyzing the pupillometry results of age- and sex-matched Parkinson’s disease patients.

Among the 192 patients with Parkinsonism who visited the outpatient clinic during the study period, data were reviewed for PD patients, while excluding 21 patients with Parkinson-plus syndrome (9 with progressive supranuclear palsy, 8 with multiple system atrophy, and 4 with corticobasal degeneration) and 4 with drug-induced parkinsonism (confirmed by dopamine transporter imaging). Among the 167 remaining PD patients, 24 de novo patients and 11 with ocular or systemic diseases were excluded, and a total of 132 patients were finally analyzed (Fig. 1).

Among them, 100 patients (75.8%) were classified as early stage and 32 (24.2%) as late stage. Patients with late stage PD were older, had a longer duration of disease, and had a higher LEDD than those with early stage PD. There was no difference between the two groups in the proportion of patients using anticholinergics. In the pupillometry results, the late stage group had smaller pupil size and slower constriction velocity and maximum constriction velocity than the early stage group (Supplementary Table S1).

### After propensity score matching

**Figure 2:**
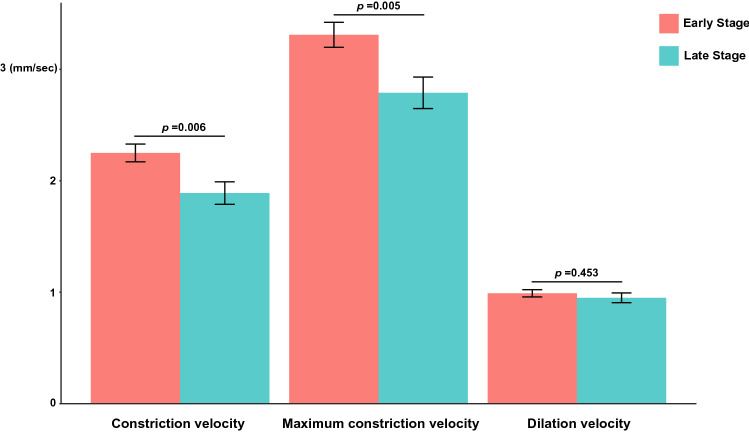
Comparison of constriction velocity, maximum constriction velocity, and dilation velocity between the early and the late stage groups. Constriction velocity and maximum constriction velocity decreased significantly in the late stage group. However, dilation velocity was similar between the two groups.

After 2:1 propensity score matching, there were 64 patients in the early stage and 32 patients in the late stage group. After matching, the age difference between the two groups decreased from 7 to 2 years, and the statistical differences in age between the two groups also disappeared. The late stage group had a longer disease duration (4 [2–7] years vs. 6 [4–9] years, *P* = 0.007) and a higher LEDD (387 ± 190 mg vs. 642 ± 296 mg, *P* < 0.001) than the early stage group. Constriction velocity (2.25 ± 0.64 mm/s vs. 1.89 ± 0.57 mm/s, *P* = 0.006) and maximum constriction velocity (3.31 ± 0.90 mm/s vs. 2.79 ± 0.80 mm/s, *P* = 0.005) were significantly lower in the late stage group than in the early stage group (Fig. 2).

However, baseline pupil size, minimum diameter, constriction latency, and dilation velocity were not significantly different between the two groups (Table 1).

**Table 1:** Characteristics of patients according to the HY stage.

	Early stage (n = 64)	Late stage (n = 32)	*P*
**Demographics**			
Age	71.8 ± 6.5	73.8 ± 7.1	0.182
Sex, woman	33 (51.6)	17 (53.1)	> 0.999
**Parkinson disease**			
HY stage	1.78 ± 0.42	3.50 ± 0.67	< 0.001
Disease duration, year	4 [2–7]	6 [4–9]	0.007
LEDD, mg	387 ± 190	642 ± 296	< 0.001
**Use of anticholinergics**	11 (17.2)	3 (9.4)	0.373
Trihexyphenidyl	5 (7.8)	1 (3.1)	
Procyclidine	6 (9.4)	2 (6.3)	
**Pupillometry**			
NPi	4.31 ± 0.32	4.29 ± 0.35	0.760
Size, mm	3.84 ± 0.67	3.62 ± 0.71	0.148
Minimum diameter, mm	2.66 ± 0.41	2.59 ± 0.47	0.445
Percent change, %	30.2 ± 5.4	28.1 ± 5.4	0.079
Constriction velocity, mm/s	2.25 ± 0.64	1.89 ± 0.57	0.006
Maximum constriction velocity, mm/s	3.31 ± 0.90	2.79 ± 0.80	0.005
Latency of constriction, s	0.26 ± 0.03	0.25 ± 0.03	0.478
Dilation velocity, mm/s	0.99 ± 0.26	0.95 ± 0.25	0.453

Values are presented as n (%), mean ± standard deviation or median [interquartile range].

*HY* Hoehn and Yahr, *LEDD* Levodopa equivalent daily dose, *NPi* Neurological pupil index.

## Discussion

In this study, we observed that PLR changes with the progression of disease stage in PD patients. Constriction velocity and maximum constriction velocity were significantly affected by the progression of PD, but there were no significant differences in variables such as minimum diameter and dilation velocities. The baseline pupil size was smaller in the late stage group, than in the early stage group, but after adjusting for age, the size difference disappeared, indicating that this difference may have been caused by age-related decrease in pupil size^11^. Regarding administration of systemic anticholinergic drugs, which is similar between two groups, and previous study also reported that the administration of these drugs did not affect the PLR^12^.

It is well known that autonomic dysfunction occurs in PD^13^. PLR is a phenomenon that is regulated by a typical automatic function involving both the sympathetic and parasympathetic pathways. Using the pupillometer, various parameters related to the PLR can be objectively measured. It is possible to determine autonomic dysfunction in an inexpensive, simple, and non-invasive way. Previous studies have reported that the constriction velocity in PD patients is lower than that in control groups, and there is a relationship between autonomic functions and PLR rate changes^6,14,15^. However, little is known about whether the PLR is affected by disease progression in PD patients, and which parameters are affected.

Pupil constrictions are mainly related to the parasympathetic nervous system, wherein afferent light stimuli are transmitted to the pretectal neurons through retinal ganglia; these pretectal neurons are projected either ipsilateral or contralateral, to the Edinger-Westphal nucleus (EWN). The preganglionic parasympathetic fiber from EWN forms a synapse in the ciliary ganglion through the oculomotor nerve, and postganglionic parasympathetic neurons secrete acetylcholine at the neuromuscular junction, resulting in pupil constriction^4^. Pupil dilation occurs because of the following mechanisms: the sympathetic neuron from the reticular activation system located in the brainstem inhibits the preganglionic parasympathetic neuron in the EWN, and it synapses with the preganglionic neuron in the ciliospinal center of Budge at C8–T1. Finally, the contraction of the iris dilator muscle is caused by noradrenaline released from the postganglionic neuron, the long ciliary nerve^16^. Pupillary abnormality with central parasympathetic involvement are well known^17^, and pathologically, Lewy body accumulation and neuronal losses in the EWN are frequently reported^18^. In addition, there are reports that the involvement of the striatal dopaminergic receptors is related to parasympathetic system dysfunction^19,20^.

In our study, we observed a decrease in constriction velocity and maximum constriction velocity in a group with severe motor symptoms of PD. Constriction velocity and maximum constriction velocity are parameters involving the parasympathetic system, and changes in these parameters suggest that in patients with PD, the abnormalities of the PLR are mainly caused by parasympathetic autonomic dysfunctions. Among the pupillometeric parameters, maximum constriction velocity is considered to be the most robust measure for detecting parasympathetic dysfunction^21^.

In contrast, there was no significant difference in dilation velocity or minimum diameter between both the early stage and late stage PD patients. In previous studies, there was no difference in pupil diameter between PD and the control group after dark adaptation were applied^22–24^. The PD group had longer latency and contraction times together with reduced contraction amplitudes, than the control group^22,25^, and the peak constriction velocity of the PD group decreased than that of control group^26^. Pupillometry studies conducted in AD patients have reported that their dilation and constriction velocities were significantly slower than healthy control group, and this finding is known to be associated with degenerative changes in the locus coeruleus (LC) in patients with AD^27^. Since it is well known that neurodegenerative changes in LC appear in both AD and PD patients^28^, further research is needed to discern why the sympathetic dysfunction measured by the PLR in advanced PD patients has not been identified. One explanation is that dilation velocity of all patients in our study was measured in the “ON” state, and taking dopaminergics may have an effect on the sympathetic nervous system, thereby offsetting the progression of PD on the dilation velocity^29,30^.

This study had several limitations. First, it had a cross-sectional design and involved retrospective analyses. Although all patients who presented to our hospital during the study period were analyzed, there may have been a bias caused by the retrospective study design. Changes in the pupillometry findings of PD patients were not measured according to their disease progression from their initial diagnosis stage. Second, because this study was conducted in a single university hospital in Korea, the results should be generalized with caution. Third, the method employed to measure patients’ motor symptoms was limited to determining their HY stages. Information on the patients’ UPDRS scores and other motor complications remains insufficient, and there was no distinction between patients with the tremor dominant type of PD and those with the postural instability-gait disturbance type. However, considering the previous finding that MDS-UPDRS increases in proportion to the HY stage, there seems to be no major problems in reflecting the disease severity of PD based on the HY stage^31^. Fourth, all motor symptoms were evaluated in the ON state, and there are no evaluation results for the off state. Fifth, autonomic function tests other than pupillometry were not performed. Hence, based solely on the results of this study, it may be a quick conclusion to evaluate the changes in the autonomic nervous system according to the progression of disease in PD patients. However, previous studies have reported that the PLR may be associated with autonomic nervous system function in PD patients. Parkinsonian motor deficits are known to be different among patients with similar striatal dopamine levels^32^; therefore, it is thought that a parallel study analyzing dopamine transporter imaging is necessary to determine the extent which pupillometry results are related to dopaminergic deficits.

Nevertheless, this study has several strengths. The PLR was measured with a constant light stimulus using automated pupillometry, and the test location was constant, and the iris colors were similar due to the patients’ ethnicities being similar, thus minimizing the external effects of the test. Considering the results of previous studies showing that the PLR changes with aging, it is thought that the age-matched study design would have helped to improve the accuracy of this study. In addition, it is believed that using large sample size of patients with various stages of PD contributed to improving the accuracy of the study.

In conclusion, the parameters measured by pupillometry changed according to the motor progression of PD. In particular, constriction velocity and maximum constriction velocity decreased significantly, suggesting that pupillary parasympathetic dysfunction progresses with the progression of PD. In contrast, the factors related to sympathetic dysfunction did not change much, implying that pupillary sympathetic dysfunction advances relatively slowly even PD advanced. This study shows the possibility of observing the disease progression of PD by measuring the constriction velocity of the pupil using a pupilometer.

## Supplementary Information

Supplementary Table S1.

## Data Availability

Anonymized data and documentation from this study can be available to qualified investigators on reasonable request.
