# Supplementary material for: Analysis of pupillometer results according to disease stage in patients with Parkinson’s disease
Source: Sci Rep. 2021 Sep 9;11:17880. doi: 10.1038/s41598-021-97599-4 (PMC8429555; doi:10.1038/s41598-021-97599-4)
Supplement: Supplementary file 1 — Supplementary Table S1. [file 41598_2021_97599_MOESM1_ESM.pdf]

Analysis of pupillometer results according to disease stage in patients with Parkinson's disease

Sooyeoun You, MD, PhD,[1] Jeong-Ho Hong, MD, PhD,[1] Joonsang Yoo, MD[2]

Department of Neurology, Dongsan Medical Center, Keimyung University School of Medicine, Daegu, Republic of Korea[1]

Department of Neurology, Yongin Severance Hospital, Yonsei University College of Medicine, Yongin, Korea[2]

Supplementary Table S1. Characteristics of patients according to the HY stage (Before propensity score matching)

	Early stage (n=100)	Late stage (n=32)	*P*
Demographics			
Age	66.7 ± 10.2	73.8 ± 7.1	<0.001
Sex, woman	54 (54.0)	17 (53.1)	>0.999
Parkinson disease			
HY stage	1.77 ± 0.42	3.50 ± 0.67	<0.001
Disease duration, yr	4 (2–7)	6 (4–9)	0.006
LEDD, mg	396 ± 216	642 ± 296	<0.001
Use of anticholinergics	16 (16.0)	3 (9.4)	0.563
Pupillometry			
NPi	4.24 ± 0.38	4.29 ± 0.35	0.547
Size, mm	3.93 ± 0.65	3.62 ± 0.71	0.036
Minimum diameter, mm	2.73 ± 0.42	2.59 ± 0.47	0.132
Percent change, %	30.0 ± 5.08	28.1 ± 5.37	0.084
Constriction velocity, mm/sec	2.31 ± 0.60	1.89 ± 0.57	0.001
Maximum constriction velocity, mm/sec	3.40 ± 0.83	2.79 ± 0.80	<0.001
Latency of constriction, sec	0.25 ± 0.03	0.25 ± 0.03	0.770
Dilation velocity, mm/sec	1.03 ± 0.24	0.95 ± 0.25	0.129

Values are presented as n (%), mean ± standard deviation or median [interquartile range].

HY, Hoehn and Yahr; LEDD, Levodopa equivalent daily dose; NPi, Neurological pupil index
